# Roles of Commensal Microbiota in Pancreas Homeostasis and Pancreatic Pathologies

**DOI:** 10.1155/2015/284680

**Published:** 2015-08-06

**Authors:** Camila Leal-Lopes, Fernando J. Velloso, Julia C. Campopiano, Mari C. Sogayar, Ricardo G. Correa

**Affiliations:** ^1^Department of Biochemistry, Chemistry Institute, University of São Paulo, 05508-000 São Paulo, SP, Brazil; ^2^Cell and Molecular Therapy Center (NUCEL-NETCEM), School of Medicine, University of São Paulo, 05360-130 São Paulo, SP, Brazil; ^3^Sanford Burnham Prebys Medical Discovery Institute, La Jolla, CA 92037, USA

## Abstract

The pancreas plays a central role in metabolism, allowing ingested food to be converted and used as fuel by the cells throughout the body. On the other hand, the pancreas may be affected by devastating diseases, such as pancreatitis, pancreatic adenocarcinoma (PAC), and diabetes mellitus (DM), which generally results in a wide metabolic imbalance. The causes for the development and progression of these diseases are still controversial; therefore it is essential to better understand the underlying mechanisms which compromise the pancreatic homeostasis. The interest in the study of the commensal microbiome increased extensively in recent years, when many discoveries have illustrated its central role in both human physiology and maintenance of homeostasis. Further understanding of the involvement of the microbiome during the development of pathological conditions is critical for the improvement of new diagnostic and therapeutic approaches. In the present review, we discuss recent findings on the behavior and functions played by the microbiota in major pancreatic diseases and provide further insights into its potential roles in the maintenance of pancreatic steady-state activities.

## 1. Introduction

**Figure 1:**
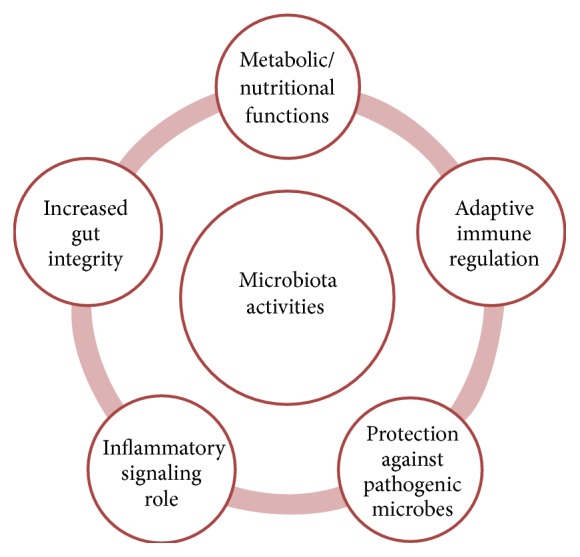
The different routes of interaction between the microbiota and the host.

The human microbiota (the ecological community of commensal, symbiotic, and pathogenic microorganisms present in our body) or microbiome (entire genome sequence of a microbial community) [1, 2] has recently emerged as an important factor in human physiology, both under homeostatic (health) and pathological conditions [3]. The microbiome is predominantly formed by bacteria but also comprises fungi, yeast, viruses, and archaea that live in our bodies, with each particular region of the body corresponding to a highly specialized niche characterized by its own microbial clusters, society dynamics, and interaction with the host tissue [4]. Remarkably, 90% of the cells in the human body are constituted by prokaryotic cells which form the microbiota [5] and participate in metabolic functions, contribute to the education of the immune system, protect against pathogenic microorganisms (Figure 1), and, through these basic functions, directly or indirectly, affect many of our physiological functions [6].

The gastrointestinal (GI) tract is certainly the greatest microbial compartment in the body, with up to 100 trillion microorganisms and over 1,000 different bacterial resident species [7, 8], and has been one of the most carefully examined ecosystems. This compartment also contains the largest surface in the human body, with the villi and microvilli of the small bowel corresponding to a total area of ~2,700 square-feet, overcoming those of the skin, lungs, nasal cavity, and sinusoids. For this reason and due to the growing number of disorders associated with microbiota unbalance (dysbiosis or dysbacteriosis), the interest of several research groups has converged to the GI microbiota and its associations with human health. Thus, extensive research has been focused on understanding the intimate relationship between the GI microbiota, diet, metabolism, and the immune system. Specifically, an increasing number of genomic-based molecular techniques, such as transcriptome, metabolome, and metagenome analyses, combined with the use of various* in vivo* models, such as germ-free mice, have expanded our current knowledge on microbiomes [9].

The interaction between host cells and a large variety of microorganisms occurs primarily through the action of pattern recognition receptors (PRRs) that compose the innate immune system. Different families of PRRs have essential roles in combating pathogens during innate and adaptive immune response, such as the Toll-like receptors (TLRs) and the cytosolic Nucleotide-binding oligomerization domain- (NOD-) like receptors (NLRs) [10]. Since these receptors recognize microorganism-associated molecular patterns (MAMPs), it is reasonable to consider their importance in the microbiota context. Due to the physiological importance and active role of TLRs and NLRs in a subset of autoimmune and proinflammatory diseases, dysregulation of microbial sensing due to functional or genetic defects has been reported to influence a series of disease outcomes, including tumorigenesis. For instance, it has been shown that lipopolysaccharides (LPS), a TLR4 agonist, and ssRNA (TLR7 and TLR8 ligands) accelerate pancreatic carcinogenesis [11, 12]. Also, genetic ablation of TLR4 [13], blockade of TLR9 [14], and TLR7 ablation in immune cells attenuate pancreatic carcinogenesis [11]. Similarly,* TLR4* and* NOD1* knockdown mice are protected from acute pancreatitis [15]. These procarcinogenic effects of microbe recognition, mediated by TLRs and NLRs, seem to involve chronic low-grade activation of the immune system and perpetuation of tumor-associated inflammation, as a result of the production of several downstream proinflammatory factors [16]. The adapter protein MyD88 (myeloid differentiation primary response gene 88) and TRIF (Toll/IL-1 receptor- (TIR-) domain-containing adapter-inducing interferon-*β*) are described as key molecules in the TLR signaling pathway that transduce the activation of the NF-*κ*B, MAPK, and IRF, stimulating the production of various cytokines and chemokines, such as TNF-*α*, IL-6, IL-12, IFN-*α*, and IFN-*β* [17]. Inhibition of either NF-*κ*B or MAPK pathways has been shown to prevent procarcinogenic effects of TLRs [12]. Stimulation of NOD1 and NOD2 also induces production of cytokines and chemokines, dependent on MAPK and NF-*κ*B signaling, whereas activation of NLRs such as NLRC4, NLRP1, NLRP3, and NLRP6 culminates in the formation of inflammasomes. As a result, further activation of caspase-1 and secretion of IL-1*β* and IL-18 mediate inflammatory processes and a distinct mechanism of programmed cell death known as pyroptosis [18]. The downstream factors in NLR signaling also seem to be necessary to keep the balance in the intestinal microbiota, since the inflammasomes have been found to contribute to the pancreatitis pathogenesis [14] and deficiency of several NLRs, caspase-1, or IL-18 led to alterations in gut microbiome and susceptibility to colorectal cancer [19].

The interfaces between the host immune system and the microbiota are frequent, intricate, and bidirectional. The immune system learns to tolerate the commensal microbiota and respond correctly to pathogens, while the microbiota instructs the immune system to work appropriately. Some studies have described the indispensable role of microbiota on maintaining the immune homeostasis by promoting the differentiation of anti-inflammatory regulatory T cells (T_REG_). T_REG_ cells have a key role in maintaining self-tolerance via the suppression of self-reactive T cells, thereby preventing autoimmune responses [20, 21]. For instance, it has been observed that different nonpathogenic* Clostridium* species are able to induce T_REG_ cells in the colon. Among the potential mechanisms involved in this T_REG_ induction, the butyrate production was shown to have an epigenetic action by controlling the* Foxp3* promoter [22, 23], besides the supply of a TGF-*β*-rich environment that also directs T_REG_ differentiation [24, 25]. Interestingly, infection by* Helicobacter pylori* has been considered as a causal factor for the development of peptic ulcer, while its participation in the induction of T_REG_ cells seems to be an important mechanism in the control of asthma [26]. In three independent epidemiologic studies, seropositivity to* H. pylori* was correlated with reduced risk of childhood-onset asthma, as well as cutaneous allergies and allergic rhinitis [27, 28]. These observations were supported by other studies on experimental murine asthma models in which the protective role of persistent early life* H. pylori* infection could be adaptively transferred using purified CD4^+^ CD25^+^ T cells from the neonatally infected mice [29]. In addition, there is indication that* H. pylori* colonization protects against common infections, including those leading to diarrheal diseases of childhood [30] and tuberculosis [31]. Further investigations have led to the development of a new method to identify bacterial strains capable of controlling T_REG_ development in human fecal microbiota [32]. Random introduction of human fecal strains in germ-free mice identified an unexpected range of bacterial strains that promoted increased numbers of T_REG_ cells in the colon, as well as strains that modulate adiposity and cecal metabolite concentrations [32]. Therefore, this microbiota-dependent T_REG_ induction is an essential mechanism for the prevention of spontaneous inflammation against commensal microbes and homeostasis conservation, as it is also important in protecting the host against pathological conditions.

Dysbiosis in the human body has been widely related to the development of several diseases. For instance, deficient/insufficient content of the normal microbiota, especially during the early development of the immune system, may lead to dysregulation of immune effector cells, accounting for changes in systemic, nonintestinal allergic conditions. In addition, alterations in the microbiota resulting from exposure to various environmental factors, including diet, toxins, drugs, and pathogens, trigger pathological conditions both inside and outside the GI tract. In fact, dietary interventions, alterations in the intestinal microbiota, and exposure to enteric pathogens regulate the development of autoimmune diabetes, wherein these modulations increase gut permeability, affect intestinal immunity, and impair regulatory mechanisms [33].

The GI tract disorders related to microbial dysbiosis include coeliac disease, irritable bowel syndrome (IBS), and inflammatory bowel disease (IBD). Extraintestinal disorders related to microbial dysbiosis comprise diseases that may affect many other organs, particularly the pancreas. Interestingly, the pancreas does not have an identified microbiome; however, it can be deeply affected by dysbiosis in the gut. Indeed, the role of the gut as a regulator of type 1 diabetes (T1D) has been suggested in animal and human studies, where changes affecting the gut microbiota modulate the incidence of diabetes. Still, the causes for the development and progression of diseases such as diabetes, pancreatitis, and pancreatic cancer are still controversial. The interest in recent years to investigate the human microbiome, in addition to the continuous findings of its participation in several aspects of human physiology, has opened new possibilities to understand the pathophysiology of various disorders. In this review, we will particularly discuss the recent advances on understanding the role of the microbiota in pancreatic diseases and present our perceptions of this important research topic.

## 2. Pancreas: Anatomy and Function

The pancreas is an abdominal organ that lies behind the stomach and is surrounded by other organs, such as the small intestine, liver, and spleen. It has a central role in metabolism, allowing ingested food to be converted and used as fuel by the cells throughout the body. The pancreas has two basic functional compartments: (1) the exocrine portion, which secretes digestive enzymes for food digestion in the intestine and (2) the endocrine portion that maintains glucose homeostasis. Both pancreatic compartments originate from the same progenitor cells in the dorsal and ventral buds of the foregut [34, 35].

The exocrine component represents 98-99% of the pancreatic mass, consisting of a highly branched, tubular, epithelial tree-like network [36]. This portion is comprised mainly of acinar, centroacinar, and ductal cells. Acinar cell clusters secrete digestive enzymes, such as amylase, in the distal ends of capped ductal branches, which are connected to a trunk-like central duct that shuttles the enzymes into the duodenum [37, 38]. Enzymes secreted by the acini, along with the bile, aid in the digestion of fats, carbohydrates, and proteins and in the absorption of nutrients. In addition to the enzymatic secretion, the exocrine portion of the pancreas is responsible for secreting water and ions into the intestine, thereby adjusting the gastric pH [39].

The endocrine portion is mainly organized into cell aggregates or islets, dispersed within the exocrine tissue, accounting for approximately 2% of the organ mass [40, 41]. Pancreatic islets monitor bloodstream glucose and secrete hormones accordingly to maintain normoglycemia [42, 43]. Islets are comprised of *β*, *α*, *δ*, *γ*, and *ε* cells, which secrete respectively, insulin, glucagon, somatostatin, pancreatic polypeptide, and ghrelin [44, 45]. In addition, islets secrete several neuropeptides and cotransmitters that mostly modulate the exocrine pancreatic function [46].

Efforts to characterize the pancreatic physiology and development have been driven, in part, by the devastating nature of pancreatic diseases, mainly exocrine disorders, such as pancreatitis and pancreatic adenocarcinoma (PAC), as well as endocrine disorders such as diabetes mellitus (DM). For instance, PAC, normally diagnosed on its later stages, offers one of the worst prognoses among cancers.

Pancreatic diseases generally result in a wide metabolic imbalance. In fact, the occurrence of local inflammation, carcinoma, or DM affects the functions of *β*-cells as glycaemia-level sensor and insulin secretor, which disrupt glucose homeostasis and the proper metabolism of tissues that rely solely on glucose as energy source (e.g., nervous system) [47]. Therefore, it is essential to understand the progression of pathological mechanisms that compromise the pancreatic function.

## 3. Microbiome and Pancreatitis

Inflammation of the pancreas (pancreatitis) is one of the most prevalent pancreatic disorders worldwide [48]. Acute cases are frequently prompted by structural blockage such as gallstones [49] or damage by alcohol consumption [50]. Chronic cases are characterized by repeated mild acute episodes of inflammation in the pancreas, leading to cell infiltration and fibrosis. Pancreatitis gives rise to widespread complications since fibrotic tissue and inflammatory infiltrates affect the exocrine pancreas, causing digestive and absorption disorders, as well as the endocrine portion, leading to diabetes. The acute pancreatic inflammation also increases intestinal permeability and bacterial overgrowth, allowing for secondary infections and endotoxemia [51, 52]. The well-established link between inflammation and carcinogenesis is reflected in the pancreatitis, since the most common cause of death in chronic patients is pancreatic cancer [53].

In most cases of acute pancreatitis, inflammation is driven by molecular sensing of tissue damage [54]. The initial injury is characteristically sterile and results in acinar cell necrosis. Intracellular contents released from damaged cells into the extracellular space serve as DAMPs (damage-associated molecular patterns) that trigger inflammation. There is increasing evidence that this sterile inflammatory response mediated through DAMPs is a key determinant of further pancreatic injury. A number of DAMPs, including high-mobility group box protein 1 (HMGB1), DNA, adenosine triphosphate, and heat shock protein 70 (Hsp70), have been shown to have a role in experimental pancreatitis [54]. Many of these DAMPs are also detectable in clinical cases of pancreatitis. HMGB1 is released by necrotic acinar cells in experimental and human disease and mediates further tissue injury and inflammation in sterile inflammatory injury through TLR4 [55]. HMGB1 is markedly elevated in the serum of patients with acute pancreatitis (AP) and also correlates with disease severity [56, 57]. Exogenous Hsp70 increases pancreatic injury in rodent models of AP through a TLR4-dependent manner, while the role of endogenous Hsp70 as potential DAMP is less clear [58]. Genetic deletion and pharmacologic antagonism have demonstrated that specific DAMP receptors, including Toll-like receptors TLR4 and TLR9, are also required for inflammation in experimental acute pancreatitis [54]. Furthermore, the direct proinflammatory role of TLR4 in the progression of caerulein or L-arginine-induced acute pancreatitis was demonstrated independently of LPS by the genetic deletion of TLR4 [13, 59]. Additionally, TLR4 and TLR9 stimulation can induce pancreatic injury in the context of a proinflammatory state. Repeated administration of TLR4 and TLR9 ligands induces pancreatic injury and inflammation in mice genetically deficient in interleukin-10 (IL-10), an anti-inflammatory cytokine known to suppress proinflammatory responses in the pancreas [60, 61]. The expression and variability of TLRs modulate the interaction with DAMPs and the resulting inflammation [62].

Other downstream DAMP-sensing components are also required for full experimental pancreatitis. For example, the cytosolic protease caspase-1, which is part of the inflammation cascade, is required for full acinar cell death and inflammation in experimental models, since its genetic deletion greatly reduces these responses [14, 63]. NLRP3 (Nucleotide-binding domain, leucine-rich repeat-containing family, pyrin domain-containing 3) is another DAMP sensor required for maximum injury in experimental AP and is expressed in tissue macrophages [14]. Interleukin-1*β* (IL-1*β*) and Interleukin-18 (IL-18) are key effector cytokines in the innate immune responses. Both are transcriptionally induced by TLR signaling and processed to their active forms by caspase-1. Blocking of IL-1*β* with specific antagonists decreases the severity of experimental acute pancreatitis [64, 65], further supporting a role for IL-1*β* in mediating pancreatic injury. Pancreas-specific overexpression of an IL-1*β* transgene resulted in chronic pancreatitis [66]. IL-18 serum levels consistently correlate with severity of pancreatic injury [67, 68], while its genetic depletion results in significantly more pancreatic injury [69], suggesting an important role in the local immune response. Other Toll-like receptors were also associated with pancreatitis, including TLR3 and TLR6, whose genetic polymorphisms are associated with the occurrence of severe pancreatitis [70]. Also, repeated stimulation of innate immunity by TLR agonists and LPS induces autoimmune pancreatitis in mice via imbalanced proinflammatory cytokines [61].

The commensal microbiota may play a role in the initial onset of pancreatic inflammation. In fact, the gut microbiota has a synergistic interplay during this inflammatory process [71]. Pancreatic damage increases intestine permeability [72] and causes ischemia and bacterial overgrowth in the gut, translocating intestinal microbiota to the pancreas which may promote secondary infections. In fact, infection of necrotic pancreatic tissue is one of the most important causes of mortality in acute pancreatitis [73, 74]. Conversely, it has also been shown that the initial onset of cerulean-driven acute pancreatitis is dependent on the activation of NOD1 in acinar cells by commensal bacteria translocated from the gut, which further induces the expression of inflammatory mediators [15].

Primary pancreatic inflammation can also be a result of an autoimmune response. Autoimmune pancreatitis (AIP) represents 4–6% of chronic pancreatitis cases and is often associated with other autoimmune diseases, particularly Sjögren's syndrome [75, 76]. The most important diagnostic feature of AIP is the elevated serum immunoglobulin G4 (IgG4) levels [77]. Patients affected by AIP frequently present antibodies against human carbonic anhydrase II (CA-II), an enzyme of the pancreatic epithelium, suggesting a role for these proteins as autoantigens in the disease [78]. It has been shown that a specific HLA-DR genotype represents a risk factor for the development of AIP [79].* Helicobacter pylori* infection has been previously associated with the other autoimmune conditions, via molecular mimicry of host structures [80]. Based on this evidence, it was proposed that gastric* Helicobacter pylori* infection could trigger AIP through molecular mimicry between human and bacterial antigens [81]. Further* in silico* evidence pointed to the significant homology between CA-II and *α*-carbonic anhydrase of* Helicobacter pylori* (HpCA). Moreover, the homologous segments contained the binding motif of the high risk HLA allele [82]. These results suggest that infection by* Helicobacter pylori* can trigger autoimmune pancreatitis in genetically predisposed subjects.

The possible participation of other microbial infections has also been suggested in the pathogenesis of AIP [83, 84]. For instance, mice inoculated with heat-killed* Escherichia coli* had marked pancreatic inflammation and fibrosis resembling human AIP pathology. Furthermore, sera from this mice presented antibodies for carbonic anhydrase [85]. Other microbial components have been suggested as molecular triggers for AIP, such as LPS [61] and TLR3 ligand double-stranded RNA (dsRNA) [86]. These components might be recognized as PAMPs by several TLRs. In rat models, pancreatic stellate cells normally express mRNAs for TLR2, TLR3, TLR4, and TLR5 [87]. Also, the TLR7 receptor, which recognizes several viral ssRNAs, is highly expressed in AIP pancreata [88, 89]. Indeed, TLR7 might participate as a key member molecule involved in the progression of autoimmune inflammation. Still, TLR7 activation might also reflect a secondary inflammatory response to ssRNA liberated by cellular damage.

Due to its involvement in the development of pancreatitis, regardless of the etiology, the inflammasome pathway might provide novel therapeutic targets by deriving antagonists of certain PRRs [54]. The peroxisome proliferator-activated receptor-*α* (PPAR-*α*) has also attracted considerable attention for its anti-inflammatory properties. Use of PPAR-*α* agonist reduced inflammation and severity in acute pancreatitis via repression of TLR2 and TLR4 mRNA [90]. Lactate administration also negatively regulates TLR induction of NLRP3, preventing activation of NF-*κ*B in macrophages, reducing the severity of acute pancreatitis [91]. Another therapeutic approach was the administration of lornoxicam in acute pancreatitis patients, who presented reduced mortality associated with reduced TLR2 and TLR4 mRNA levels in the peripheral blood mononuclear cells [92].

Nevertheless, the molecular basis for the pathogenesis associated with pancreatitis remains elusive. The deranged function of the gut mucosal barrier and the presence of enteric Gram-negative bacteria in the pancreas suggest the participation of microbiota in the development of pancreatitis. To this end, the gut apparently has a role in neutrophil priming and release of proinflammatory cytokines, both of which are important at the beginning and during propagation of inflammation and sepsis [93].

## 4. Microbiome and Pancreatic Cancer

Pancreatic cancer is the twelfth most common type of cancer (2% of the total cases) and the seventh cause of cancer deaths worldwide [94, 95]. Pancreatic cancer is not nearly as prevalent as lung or prostate cancers; however, pancreatic tumors are extremely aggressive, leading to the worst prognosis for any kind of cancer, with a five-year survival rate of ~5% [96, 97]. Both exocrine and endocrine cells of the pancreas can form cancers, but those formed by exocrine cells are described to be much more common and aggressive [98, 99]. About 95% of pancreatic cancers are adenocarcinomas, originated in gland cells [100, 101]. These cancers usually arise from ductal cells but may also develop from enzyme secreting cells, being denominated acinar cell carcinomas. Other rarer cancers of the exocrine pancreas include adenosquamous carcinomas, squamous cell carcinomas, signet ring cell carcinomas, undifferentiated carcinomas, and undifferentiated carcinomas with giant cells [102].

Tumors of the endocrine pancreas are very uncommon, making up less than 2% of all pancreatic cancers. They are known as islet cell tumors or neuroendocrine tumors (NET) [103, 104]. About 50% of tumors in islet cells are functioning tumors, maintaining hormone secretion. The exacerbated release of hormones in the bloodstream causes a metabolic imbalance, usually leading to hypo- or hyperglycemia. Functioning tumors are characterized by originating hormone-producing cell. The most common types are gastrinomas, glucagonomas, and insulinomas. Differently from most NETs, the insulinomas, which arise from *β*-cells, are the most common pancreatic endocrine tumor, accounting for 70% of NETs, with an incidence of 1 to 4 per million [105]. Insulinomas are usually benign, solitary, and intrapancreatic, with less than 10% of the cases presenting a metastatic behavior [105, 106]. The metastatic insulinomas usually spread to the liver and lymph nodes but, nevertheless, the prognosis is far better than that of patients with exocrine pancreatic cancer. Due to the low malignancy and constant production of insulin by the tumor mass, insulinomas are usually detected due the systemic metabolic alterations that follow insulin oversecretion. In fact, insulinomas are considered the commonest cause of endogenous hyperinsulinaemic hypoglycaemia (HH) in adults [107]. HH arises from many conditions that cause insulin secretion to become inappropriate for the level of blood glucose. HH is a major cause of persistent hypoglycaemia in the childhood period [108], either being caused by secondary factors such as growth retardation or being congenital, due to defects in key genes involved in regulating insulin secretion [109]. In adults, apart from an insulinoma, HH has been reported with several conditions, including insulin autoimmune syndrome and noninsulinoma pancreatogenous hypoglycaemia syndrome, and in patients with mutations on the insulin receptor [110, 111].

In the last few years, several studies have presented substantial data suggesting a role for the oral and gut microbiota in pancreatic cancer [16]. In this context, the generation of germ-free mice has been extremely valuable to better understand the influence of the microbiome in carcinogenesis. In most models, these animals are less inclined to carcinogenesis, probably due to decreased tumor-associated inflammation [112, 113]. The same profile is observed in antibiotic-treated mice that reduces the microbial load of the gut [114]. Remarkably, bowel sterilization with broad-spectrum antibiotics appears to be protective in acute pancreatitis [15]. An epidemiologic study revealed associations between specific profile of oral bacteria and the risk of pancreatitis and pancreatic cancer [115]. In this context, a decrease in the levels of* N. elongata* and* S. mitis*, with concomitant increase of* G. adiacens*, has been observed, suggesting the use of this bacterial profiling as a biomarker for pancreatitis and pancreatic cancer [115].

An underlying physiological condition in both pancreatitis and many pancreatic cancers is the inflammation of exocrine and endocrine tissues. Inflammation is a well-established condition that contributes to carcinogenesis [116, 117] and is often caused by dysbiosis of the host microbiota [112, 118, 119], which can lead to opportunistic infections by agents such as bacteria and viruses [120]. Dysbiosis may occur by infections, antibiotics, obesity, or innate immune responses and has been mechanistically linked to GI cancers [121, 122]. The link between chronic inflammation and the development of pancreatic adenocarcinoma is becoming clearer due to extensive studies [123]. Indeed, chronic pancreatitis is well established as a risk factor for the development of pancreatic cancer [50, 53, 100, 123, 124]. Additionally, the duration of pancreatitis seems to correlate positively with the predisposition of* KRAS* oncogene mutations, suggesting a possible mutagenic role for repetitive bouts of inflammation [125]. Also, in a mouse model with mutated KRas, inflammatory insults dramatically enhance the risk for pancreatic malignant transformation [126]. In another model, selective expression of endogenous* KRAS* during adulthood was only carcinogenic when followed by induction of chronic pancreatitis [127]. Furthermore, mutated KRas was shown to be hyperstimulated by LPS-driven inflammation or by the overexpression of genes in the NF-*κ*B pathway, accelerating pancreatic carcinogenesis [128, 129]. Thus, focal inflammation has been shown to potentially enhance cellular proliferation and mutagenesis, reduce adaptation to oxidative stress, promote angiogenesis, and inhibit apoptosis [130, 131].

Opportunistic microorganisms have been implicated in the pathogenesis of pancreatic diseases, including pancreatic ductal adenocarcinoma and autoimmune pancreatitis, most notably the bacteria* Helicobacter pylori* [81, 82, 132, 133] (Table 2). In recent studies, an antigenic peptide of* H. pylori* was identified in patients with autoimmune pancreatitis and pancreatic adenocarcinoma [88]. Additional data support this association presenting* H. pylori* colonization as a risk factor for pancreatic cancer [134, 135]. These microorganisms usually infect the pancreas via translocation from the gut [136]. Infection by* H. pylori* promotes upregulation of NF-*κ*B, which is constitutively activated in several types of cancers, including pancreatic cancer, and can also be induced by several types of inflammatory cytokines including IL-1*β* in pancreatic cancer [137, 138]. A potent activator of NF-*κ*B in pancreatic cancer is LPS, released from the surface of Gram-negative bacterial cell wall, providing another possible link between microorganism-driven inflammation and cancer development and progression [139]. Although accumulating evidence shows the association of microorganisms such as* H. pylori* with pancreatic ductal adenocarcinoma (PDAC), no single pathogen has been mechanistically demonstrated as causative for pancreatic cancer [16].

New techniques such as next generation sequencing and metagenomics now enable a representative evaluation of the microbiotic communities in health and disease and their dynamic interactions with their human host [140]. The role of such global shifts in the microbiome composition has not been evaluated in the context of pancreatic carcinogenesis [16]. The most plausible mechanism for a carcinogenic effect of microbiota shifts is by chronic activation of innate immunity leading to chronic inflammation. As previously stated, the microbial pattern recognition by Toll-like receptors (TLRs) is a cornerstone of innate immunity and represents one of the most powerful proinflammatory stimuli via binding of a variety of MAMPs, such as LPS and byproducts of dying cells and sterile inflammation (also denoted by damage-associated molecular patterns, DAMPs) [141]. Accumulating evidence indicates that the binding of MAMPs to specific TLRs contributes to carcinogenesis in pancreas via activation of NF-*κ*B and MAPK pathways [11, 12]. In fact, in mice pancreatic tumor models, carcinogenic progression was greatly accelerated by the administration of lipopolysaccharide (LPS), a Gram-negative bacterial cell wall component which is specifically recognized by TLR4 [12]. Furthermore, inhibition of TLR4 in the same model is protective, while blockade of MyD88 surprisingly exacerbates pancreatic inflammation and malignant progression. The protumorigenic and inflammatory effects of MyD88 inhibition are mediated by dendritic cells (DCs), which induce pancreatic antigen-restricted Th2-deviated CD4^+^ T cells and promote the transition from pancreatitis to carcinoma [12]. Also, in an acute colitis model, constitutively activated epithelial-derived TLR4 in the gut drives tumorigenesis, together with enhanced expression of inflammatory mediators and increased neutrophil infiltration [142].

Pancreatic ductal adenocarcinoma (PDAC) has no clear early symptoms or screening methods, being normally diagnosed only in more advanced stages [143]. Diagnostic tools based on microbiota profiling could significantly improve survival rates associated with PDAC [115]. The oral cavity is a large reservoir of bacteria composed of more than 700 species or phylotypes [144]. Profiling of the saliva microbiome revealed that the microbial composition shifts significantly when comparing healthy individuals to patients with PDAC or even with other pancreatic diseases [115]. The validated bacterial signatures were associated with pancreatic cancer and pancreatitis, providing not only a possible link between the microbiota and pancreatic diseases but also a tentative source of biomarkers for diagnostics.

It is not fully understood whether there is a causative correlation between the abundance of oral microbiota and pancreatic carcinogenesis. Still, the oral microbiota could reflect the systemic alterations prompted by an early stage carcinoma. On the other hand, inflammatory oral diseases caused by bacteria, such as periodontitis, could trigger carcinogenesis. Several prospective studies have recently shown positive associations between the incidence of pancreatic cancer and either inflammatory periodontal disease [145, 146] or tooth loss [147]. In the aforementioned studies,* H. pylori* was correlated with periodontal disease but not with tooth loss. The populations of oral microorganisms, commonly associated with periodontal disease [148, 149], such as* N. elongate*,* S. mitis*,* G. adiacens*, and* P. gingivalis* are significantly altered in patients with pancreatic cancer relative to noncancer subjects [115, 145, 150]. In those studies, the population of* S. mitis* was decreased. This bacterium was shown to have a protective role against cariogenic pathogens [151], which may allow for the overgrowth of* G. adiacens*. The latter may spread systemically, as has been observed in cases of septicaemia associated with systemic inflammation [152, 153]. Also,* P. gingivalis* was shown to accelerate the progression of atherosclerosis, an inflammatory disease, by induction of host innate immunity via activation of TLR2 [154, 155]. These correlations further support the idea of systemic inflammation contributing to the progression of pancreatic diseases [115].

## 5. Microbiome and Diabetes

Diabetes mellitus (DM) belongs to a class of metabolic disorders, characterized by impairment of the insulin regulatory activity due to combined deficiency in hormone synthesis, secretion, and activity itself. According to the International Diabetes Federation, the number of people worldwide suffering from DM will increase from 387 million in 2014 to 592 million in 2035, suggesting that for every ten people at least one will develop diabetes [156].

DM may be generally classified as type 2 (T2D) and type 1 (T1D), according to the mechanisms of incidence of the disease [157]. T2D is characterized by resistance to insulin activity and partial loss of insulin production. Genetic predisposition may influence the development of T2D, but there are marked risk factors such as obesity, advanced age, lack of physical activity, hypertension, and dyslipidemia. The disease is related to a state of chronic low-grade inflammation, mainly due to a proinflammatory state caused by overnutrition (obesity) through oxidative stress and higher concentrations of inflammatory mediators (mainly TNF-*α* and IL-6, known to be expressed by adipocytes) [158].

T1D is a disorder characterized by loss of insulin secretory capacity due to destruction of insulin-producing pancreatic *β*-cells through an autoimmune process, which may begin early in childhood, often before three years of age, leading the disease to be diagnosed primarily in children and teenagers [159]. This process involves several components of both the innate and adaptive immune systems, being primarily mediated by the action of T lymphocytes. Normally, CD8^+^ T lymphocytes, also known as cytotoxic T cells, recognize and kill tumorigenic or infected cells. Islets-infiltrated CD8^+^ T cells were shown to have exclusive specificity towards islet autoantigens, which proves their autoreactive nature [160]. The islet-specific autoreactive T cells that mediate the destruction of *β*-cells in T1D are not exclusively observed in patients with T1D but are also detectable in individuals without diabetes mellitus or any other autoimmune disease [161]. However, in individuals without diabetes mellitus, these pathogenic T cells are controlled by mechanisms of peripheral tolerance, including naturally occurring systems of immune modulation and the action of T_REG_ cells.

Diabetes-related autoantibodies are normally detected before the onset of clinical symptoms [162]. The main antigens described so far are insulin/proinsulin itself, glutamic acid decarboxylase (GAD65), tyrosine phosphatase-like protein IA-2, islet-specific glucose-6-phosphatase catalytic subunit-related protein (IGRP), and zinc transporter 8 (ZnT8) [163]. The detection of related autoantibodies may be used to predict the emergence of the pathological condition in apparently healthy individuals [164].

Diabetes-related autoantibodies are secondary factors for the development of the disease [165]. Molecules from HLA (Human Leukocyte Antigen) class I and class II are involved in the T cell repertoire selection during maturation of the immune system in the thymus, as well as activation and regulation of the adaptive immune response [166]. HLA gene variants are well established as primary determinants of genetic susceptibility to T1D, representing 50–60% of the total hereditary risk related to the disease [167]. Other 40 non-HLA genes were established as highly related to the development of T1D [168, 169]. Although many genes have been related to predisposition to diabetes, less than 10% of those with genetic susceptibility progress to clinical disease [161], implicating that additional factors may be required for initiating and driving the disorder [170]. Since islet-specific autoreactive T cells are detectable in individuals without diabetes mellitus and genetic susceptibility is not enough to predict the development of the disease, it is possible that environmental factors are key triggers to unbalance the equilibrium between autoreactive T cells and the mechanisms of peripheral tolerance, leading to the existence of subjects with high susceptibility but with or without DM.

The seroconversion to autoantibody positivity in T1D is preceded by inflammation of the *β*-cell mass, a state known as insulitis [171, 172]. The factors inducing such a proinflammatory state are poorly defined but may be related to chronic viral infection in the pancreatic islets, dietary factors, and intestinal inflammation due to changes in the gut microbiome alone or in combination [173].

The “hygiene hypothesis” suggests that the immune system has evolved to protect the body from all kinds of infections and that the interaction with pathogenic agents is an important way to modulate the immune system and promote self-tolerance [174]. Some studies have demonstrated that exposure to bacterial antigen or infection (mainly by coxsackie virus A, coxsackie virus B, echovirus, or enterovirus species) in the neonatal period prevents DM [175, 176], supporting the notion that immunostimulation can benefit the maturation of the postnatal immune system. The nonobese diabetic (NOD) mouse model had delayed onset and reduced incidence of diabetes when the intestinal microbiota was populated with a Gram-positive aerobic spore-forming rod (*Bacillus cereus*), compared to NOD mice maintained under germ-free conditions [177]. In addition, the data suggest that germ-free NOD mice have reduced glycemic control and deregulated immunologic and metabolic responses, with higher levels of cytokines known to influence diabetes progression in NOD mice (IFN-*γ* and IL-12), promoting an inflammatory state [178]. These data support the notion that intestinal microbiota may have a role in the development of autoimmune diabetes.

A main question still resides in understanding the mechanism through which the intestinal microbiota could specifically affect tissue inflammation in DM. In this respect, an important observation is that intestinal phagocytes, such as DCs and macrophages, capture bacterial intestinal antigens and transfer them into lysosomes for degradation [179]. This provides a direct cellular link between the intestinal microbiota and the host, a process known as bacterial translocation.

Some recent work pointed out the role of TLRs in T1D pathogenesis. It is suggested that TLRs exert their influence on the development of T1D through the modulation of immune responses following *β*-cell destruction [180], but the exact mechanism still remains elusive. Pathogen-free NOD mice lacking MyD88 do not develop T1D [181]. The MyD88 adaptor protein is used by multiple TLRs (except TLR4 and TLR3, acting through TRIF and other proteins) [182]. The* MyD88* knockout (KO) effect on diabetes is dependent upon commensal microbes since germ-free MyD88-negative NOD mice develop robust diabetes. It has been found that TLR2, TLR3, and TLR4 are dispensable for development of T1D when individually deleted, in contrast to the effect of complete protection from diabetes associated with loss of MyD88. These findings suggested that signaling through receptors that use the MyD88 adaptor is critical for T1D development and that the autoimmune T cells would probably be affected systemically in* MyD88* KO NOD mice.

Pancreatic *β*-cells express significant levels of TLR4 which render them sensitive to LPS [183, 184]. This effect can impair insulin gene expression in human islets [185], in a TLR4-dependent manner, via NF-*κ*B signaling and involving decreased* PDX-1* and* MafA* mRNA levels in pancreatic islets. PDX-1 and MafA are transcription factors which bind to specific cis-acting DNA elements present on the proximal region of the insulin promoter and activate transcription in a coordinated manner [186], thus suggesting a mechanism by which the gut microbiota might affect pancreatic *β*-cell function.

Pancreatic *β*-cells also express significant levels of TLR2 [184], another receptor which is able to recognize bacterial LPS. TLR2 expression is induced by LPS [187] and it has been proposed that upregulation of TLR2 by low levels of bacterial products can contribute to the mechanisms by which the immune system increases its response to an infection, with a probable amplification of TLR4 signaling in response to LPS [185]. Under germ-free conditions, TLR2-deficient mice are protected from diet-induced insulin resistance [188]. In contrast, TLR2 KO mice kept in a non-germ-free facility develop a phenotype which is reminiscent of metabolic syndrome, characterized by differences in the gut microbiota, with an increase in Firmicutes and a slight increase in Bacteroidetes when compared to controls [188]. Microbiota rich in Firmicutes is related to increased capacity for energy harvesting from the diet [189], explaining obesity, while Bacteroidetes is linked to an improvement in the gut barrier function and to reduced levels of LPS [190, 191]. These changes in gut microbiota were accompanied by an increase in LPS absorption and insulin resistance. The increase in LPS circulating levels caused activation of TLR4, induced endoplasmic reticulum (ER) stress, and JNK (c-Jun N-terminal kinase) activation, but no activation of the canonical NF-*κ*B pathway. There was also increased insulin receptor substrate- (IRS-) 1 serine 307 phosphorylation in the liver, muscle, and adipose tissue, leading to a reduction in insulin sensitivity and signaling, conferring the phenotype observed in the TLR2 KO mice. This sequence of events was reproduced in wild-type (WT) mice by microbiota transplantation and was also reversed by antibiotics (ampicillin, metronidazole, and neomycin in drinking water) [188]. Adding to these findings, the intestinal microbiota across subjects with T2D showed an apparent enrichment of bacteria belonging to the phyla Bacteroidetes and Proteobacteria, both of which are LPS-containing Gram-negative bacteria [192].

Further cause-consequence relationships are still to be considered in T1D patients with respect to microbiome variations. The microbiota composition of children who are highly prone to develop T1D, prior to the appearance of the first islet autoantibodies, showed variations when compared to normal controls [193]. The species* Bacteroides dorei* and* Bacteroides vulgatus* were more represented in the microbiota of children who developed T1D, suggesting that early changes in the microbiome may be useful for predicting T1D autoimmunity and that these changes occur prior to the first autoimmunity signals [194].

Metagenomic analysis of stool samples, collected from subjects with T1D, reveals a lower proportion of butyrate-producing and mucin-degrading bacteria (*Prevotella* and* Akkermansia*, resp.), while those bacteria that produce short chain fatty acids other than butyrate were elevated in T1D cases [195]. Children with *β*-cell autoimmunity have shown decreased butyrate-producing bacteria and enhancement in the count of Bacteroidetes members in fecal microbiota, which could explain the typical alterations in gut barrier, tolerance disruption, and inflammation in T1D [193]. Butyrate is known as an anti-inflammatory short chain fatty acid that contributes to colon health [196, 197], decreases bacterial transport across metabolically stressed epithelia [198], improves the intestinal barrier by increasing tight junction assembly [199], and induces mucin synthesis [200, 201]. Mucin is a glycoprotein made by the host that maintains the integrity of the gut epithelium. Taken together, this suggests that a combination of butyrate-producing bacteria in a healthy gut induces a sufficient amount of mucin synthesis to maintain gut integrity and prevents the development of diabetes. This mechanism could solidify the explanation on how genetic susceptibility can be overcome by environmental factors to prevent the development of the disease.

Using the NOD mouse model for DM, it was found that neonatal treatment with vancomycin, a glycopeptide antibiotic specifically directed against Gram-positive bacteria, lowered the incidence of the disease [159]. Bacteriological examination of the gut microbiota composition revealed that vancomycin depleted many major genera of Gram-positive and Gram-negative microbes while, interestingly, one single species,* Akkermansia muciniphila*, became dominant, reinforcing the idea that the mucolytic bacterium* A. muciniphila* plays a protective role in autoimmune diabetes development, particularly during infancy. Rats with streptozotocin-induced T1D phenotype also experienced changes in the gut microbiome, and the treatment with insulin also induced changes in the gut population [202]. The diabetic state was characterized by a massive increase in* Klebsiella*, one of the most common Gram-negative bacteria that cause severe intestinal inflammation in humans [203, 204]. The mucosal inflammation results in a leaky epithelium, allows easier passage of bacteria through the intestinal epithelium, and disturbs the intestinal immunology, a critical element in the development of the autoimmune T1D [173, 205]. The insulin treatment significantly increased the microbial diversity and practically eliminated the genus* Klebsiella* [202].

The diet may also have a role in modulating the intestinal microbiome, leading to changes in the pathological condition. For instance, when the NOD mouse model was exposed to neutral or acidified water, the change to acid liquids dramatically altered the intestinal microbiome, increased the presence of T_REG_ cells, and lowered the incidence of diabetes, suggesting that early dietary manipulation of intestinal microbiota may be a novel mechanism to delay T1D onset in genetically predisposed individuals [206]. NOD mice were also challenged to pro- and antidiabetogenic effects of gluten-containing and gluten-free diets, respectively. The group fed with gluten had higher incidence of hyperglycemia and lower presence of T_REG_ cells [207]. When the fecal microbiomes were compared,* Bifidobacterium* (probiotic strain that regulates host immune and inflammatory responses [208]),* Tannerella* (strain associated with oral infections such as periodontal disease [209]), and* Barnesiella* (strain that regulates the amount of immune-regulatory cells [210, 211]) species were increased in the group fed with gluten, whereas* Akkermansia* species (related to obesity reversal in rats [212] and, as mentioned, a mucin-degrading bacteria) was increased in the gluten-free group. Adding back gluten to the gluten-free diet reversed its antidiabetogenic effect, reducing* Akkermansia* and increasing* Bifidobacterium*,* Tannerella*, and* Barnesiella* species, suggesting that dietary gluten could modulate the incidence of T1D by changing the gut microbiome and also reinforcing the role of* Akkermansia* species in the protection from the disease [207].

Probiotic bacteria (mainly* Lactobacillus* or* Bifidobacterium*) inhibit the growth of pathogenic bacteria by acidifying the gut lumen, competing for nutrients and producing antimicrobial substances [213]. Ingestion of live probiotic cultures may alter gut microbiota in a beneficial manner, lowering circulating endotoxin levels and reducing inflammation [214]. Treatment with the probiotic strain* Bifidobacterium animalis* subsp.* lactis 420* improves the overall inflammatory and metabolic status in a mouse model of T2D [215]. Biobreeding diabetes prone (BB-DP) rats fed after weaning with* Lactobacillus johnsonii* developed T1D at a protracted rate, with a decrease in the native microbiota, host mucosal proteins, host oxidative stress response, and low levels of the proinflammatory cytokine IFN-*γ* [216]. These data support the idea that diet may have a role in modulating the intestinal microbiome and that diet modulation can lead to changes in the pathological condition.

The oral microbiome has also been related to DM. Both T1D and T2D have been associated with increased severity of periodontal disease [217]. It has been proposed that the inflamed periodontium may act as an endocrine-like source of inflammatory mediators such as TNF-*α*, IL-1, and IL-6, which can subsequently increase insulin resistance. In fact, evidence shows that there is a change in oral microbiome of patients with DM [218, 219], but its causal (or consequential) role requires a better delineation.

## 6. Conclusions and Future Perspectives

A considerable body of evidence accrued over the years, based on clinical data and experimental* in vivo* models, shows a clear correlation between changes in the commensal microbiota and the occurrence of different pancreatic diseases. Initial studies combining biochemistry, microbial biology, and molecular approaches have described the constituents of the gut microbiome, in healthy and in particular diseased states, their niches, and respective physiological roles. However, sustained research in the microbiome field is still necessary to explain whether microbial dysbiosis is the cause or the effect of diverse pathologies and, based on this, eventually provide options for clinical intervention.

The establishment and validation of an increasing number of microbial signatures are expected to translate into the early diagnosis of pathologies at particular disease states. The body of evidence pointed to in this review suggests that, so far, nonredundant microbiota changes induce particular pathological conditions (for instance, no overlap between microbial alterations causing diabetes and pancreatitis was annotated). In fact, for diabetes, a subset of the microbiota may be applied as a marker for the disease initiation and may be also employed for diagnosis [194, 220]. Changes in the microbiota coupled to increased* Bacteroides* sp. are reported even before seroconversion to autoantibody positivity. The increase in* Bacteroides* is reported in subjects with high genetic susceptibility to develop T1D who progress to the disease, but not in those who do not develop T1D (Table 1).

On the therapeutics front, administration of prebiotics and antibiotics, dietary modification, targeting of microbe biochemical pathways, and fecal microbiota transplantation (FMT) may be used in both pancreatic disease treatment and prevention. Probiotics may prevent excessive pathogen growth [221] and constitutive activation of NF-*κ*B via immunoglobulin secretion [222, 223]. However, their use is still controversial since studies in animal models [224, 225] and also in humans [226] have presented conflicting results for the outcome of pancreatitis. Administration of prophylactic antibiotics in the setting of acute pancreatitis is also contradictory since several meta-analyses of control trials associated their use with a lower mortality rate [227], while others found no preventive effects towards pancreatitis [228].

Other approaches focused on intervention of the regulatory pathways that control inflammation in innate immunity. Indeed, inhibition of cyclooxygenases by nonsteroidal anti-inflammatory drugs and COX2-specific inhibitors has been investigated in multiple studies, resulting in a decreased risk of pancreatic cancer [229, 230] and pancreatitis associated with TLR inhibition [92, 231]. Specifically, for pancreatic neuroendocrine tumors, biotherapy using somatostatin analogs to directly inhibit the TLR4-dependent NF-*κ*B [232] also showed promising results [233]. Microbial byproducts, such as butyrate, were also used to control inflammation via induction of colonic T_REG_ cells in mice [23]. Conversely, potent activation of innate immunity may convert tumor tolerance into antitumor immune response, so that intense activation of Toll-like receptors may lead to protective effects. Indeed, high doses of TLR and NOD-like receptor (NLR) agonists are associated with antitumor effects [234].

It is also worth noting that disturbances in the gut microbiota might affect not only disease progression but also responses to the treatment. Several chemotherapeutic drugs rely not only on stimulating an antitumor immune response, but also on modifying the composition of the gut microbiome and promoting the translocation of Gram-positive bacteria that, in turn, prime T cells which are necessary for the immune-mediated effects of chemotherapy. Administration of antibiotics, often required by patients undergoing chemotherapy, might reduce the immune response and render tumors resistant to these immune-modulating drugs [235, 236].

Modulation of the microbiota may also be used in T1D treatment. As listed in Table 1, the treatment with antibiotics or probiotics induces changes in microbiota that lead to improvement of the pathological condition [216, 237]. The diet may also have a role in modulating the intestinal microbiome leading to changes in the pathological condition [206, 207].

Additional knowledge is still necessary to sustain the safety and consistency of the experimental results to ensure the development of better defined and safer microbial therapeutic procedures. However, it is clear that manipulation of the microbiome may be employed to better understand and further diagnose and treat diverse pancreatic diseases. In the light of the data here discussed, it appears essential that we realize the importance of the human microbiome in pancreas homeostasis and its adaptive measures in response to both physiological and pathological conditions. Altogether, the current body of evidences opens new avenues in the search for new disease markers and targeted therapies for pancreatic maladies in the coming years.

## Figures and Tables

**Table 1:** Microbial species implicated in diabetes. A current list of the characterized microbes is shown, which includes the experimental models that were evaluated and the observed effects according to each microbial species. The respective bibliographies are also listed (last column).

Microbial species	Experimental model	Effects	References
*Bacteroides *sp.	BB-DP/BB-DR rats	Increase in rats that develop T1D over time	[238]
	Children positive to T1D autoimmune process	More abundant in case, secreting short chain fatty acids that do not induce mucin synthesis	[195]
	Meconium from children delivered by mothers with different diabetes status	Higher incidence in the meconium of children of diabetic mothers	[239]
	Fecal samples of children with beta-cell autoimmunity	Increased in fecal sample of children with beta-cell autoimmunity	[220]
	Rats with streptozotocin-induced diabetes	Increased in cases	[202]
	NOD mice	Increased after neutral water consumption with increase in diabetes incidence	[206]
	TLR2 knockout (KO) mice	Loss of TLR2 in mice results in a phenotype reminiscent of metabolic syndrome with an increase in *Bacteroides *	[188]

*Bacteroides dorei *and* Bacteroides* *vulgatus *	Stool samples from children susceptible to T1D	Higher in cases compared to controls prior to seroconversion	[193]

*Lactobacillus *strains	BB-DP/BB-DR rats	Higher incidence in DM-resistant models	[240]

*Lactobacillus johnsonii N6.2 *	BB-DP/BB-DR rats	Mitigates the development of type 1 diabetes	[216]

*Bifidobacterium adolescentis *and* Bifidobacterium pseudocatenulatum *	Fecal sample of children with b-cell autoimmunity	Decreased in fecal sample of children with beta-cell autoimmunity	[220]

*Bifidobacterium *strains	BB-DP/BB-DR rats	Higher incidence in DM-resistant models	[240]
	Mice high-fat diet-induced diabetes model	Treatment with the probiotic strain decreased bacterial translocation process from intestine towards tissue in model of high-fat diet-induced diabetes	[215]
	NOD mice	Gluten-free diet lowered the incidence of diabetes and increased this bacterial population	[207]

*Pseudobutyrivibrio *strains	BB-DP/BB-DR rats	Higher incidence in DM-resistant models	[240]

*Pontibacillus (halophilic genus) *	BB-DP/BB-DR rats	Higher incidence in DM-prone models	[240]

*Clostridium *genus*: Clostridium aldrichii Clostridium fimetarium Clostridium nexile Clostridium orbiscindens *	T2D patients	Reduced in cases	[192]

*Clostridium hylemonae *	BB-DP/BB-DR rats	Higher incidence in DM-resistant models	[240]

*Prevotella *genera	Children positive to T1D autoimmune process	More abundant in controls; synthetizing mucin	[195]

*Akkermansia *genera*: Akkermansia muciniphila *	Children positive to T1D autoimmune process	More abundant in controls; synthesizing mucin	[195]
	NOD mice	Vancomycin treatment increased the incidence of the species and lowered the incidence of DM	[237]
	NOD mice	Gluten-free diet lowered the incidence of diabetes and increased this bacterial population	[207]

*Veillonella *	Children positive to T1D autoimmune process	More abundant in case, secreting short chain fatty acids that do not induce mucin synthesis	[195]

*Alistipes *	Children positive to T1D autoimmune process	More abundant in case, secreting short chain fatty acids that do not induce mucin synthesis	[195]

*Bifidobacterium *	NOD mice	Gluten-containing diet increased the incidence of diabetes and increased this bacterial population	[207]

*Tannerella *	NOD mice	Gluten-free diet lowered the incidence of diabetes and increased this bacterial population	[207]

*Barnesiella *	NOD mice	Gluten-free diet lowered the incidence of diabetes and increased this bacterial population	[207]

Firmicutes phylum	Rats with streptozotocin-induced diabetes;	Increased in cases	[202]
	NOD mice	Decreased after neutral water consumption with increase of diabetes incidence	[206]
	T2D patients	Reduced in cases	[192]
	TLR2 knockout (KO) mice	Loss of TLR2 in mice results in a phenotype reminiscent of metabolic syndrome with an increase in Firmicutes	[188]

Human Firmicute strain CO19	Children with high genetic risk for T1D	Higher incidence in controls	[195]

*Klebsiella *	Rats with streptozotocin-induced diabetes	The diabetic state was characterized by a massive increase in *Klebsiella *	[202]

**Table 2:** Microbial species implicated in pancreatic cancer and pancreatitis. A current list of the characterized microbes is shown, which includes the experimental models that were evaluated and the observed effects according to each microbial species. The respective bibliographies are also listed (last column).

Microbial species	Experimental model	Effects	References
*N. elongata *	Saliva samples of PDAC and pancreatitis patients	Decreased in cases	[115, 145]

*S. mitis *	Saliva samples of PDAC and pancreatitis patients	Decreased in cases	[115, 145]

*G. adiacens *	Saliva samples of PDAC and pancreatitis patients	Increased in cases	[115, 145]

*P. gingivalis *	Blood samples of PDAC patients	High levels of antibodies against this species confer higher risk of pancreatic cancer	[150]

*Commensal oral bacteria *	Blood samples of PDAC patients	High levels of antibodies against this group confer higher risk of pancreatic cancer	[150]

*H. pylori *	Blood samples of patients with PDAC, gastric cancer, colorectal cancer, and controls	Pancreatic cancer cases had equal risk of *H. pylori* seropositivity as gastric cancer cases and higher risk than colorectal cancer cases and control	[241]
	Blood samples of smokers, pancreatic cancer cases, and controls	Patients with exocrine pancreatic cancer had higher rates of seroprevalence for *H. pylori *	[242]
	Blood samples of smokers, exocrine pancreatic cancer patients, and controls	*H. pylori* antigens were not associated with development of pancreatic cancer	[135]
	Blood sample of newly diagnosed PDAC cases and controls	Colonization by *H. pylori* associated with higher risk of pancreatic cancer, especially for individuals with non-O blood types	[243]
	Human PDAC cell lines	Increased activities of proliferation factors NF-*κ*B, AP-1, and SRE, and secreted higher levels of IL-8 and VEGF	[244]

LPS	Cerulein-induced pancreatitis	LPS synergizes with cerulean to induce severe acute pancreatitis	[59]
	L-Arginine-induced pancreatitis	Genetic ablation of TLR4 or CD14 mitigates acute pancreatitis	[13]
	P48^+/Cre^; LsL-KRas^G12d/+^	LPS accelerates pancreatic carcinogenesis, TLR4 and TRIF blockade attenuate carcinogenesis, and MyD88 blockade exacerbates carcinogenesis	[12]
	Ela-CreERT; LsL-KRas^G12d/+^	LPS synergizes with KRas mutation in acinar cells to induce pancreatitis and accelerate pancreatic carcinogenesis	[128]

## Abbreviations

AIP: Autoimmune pancreatitis
AP: Acute pancreatitis
BB-DP: Biobreeding diabetes prone
CD: Cluster of differentiation
DAMP: Damage-associated molecular pattern
DC: Dendritic cells
DM: Diabetes mellitus
dsRNA: Double-stranded RNA
ER: Endoplasmic reticulum
Foxp3: Forkhead box P3
GAD65: Glutamic acid decarboxylase
GI: Gastrointestinal
HH: Hyperinsulinaemic hypoglycaemia
HLA: Human Leukocyte Antigen
HMGB1: High-mobility group box protein 1
Hsp70: Heat shock protein 70
IFN: Interferon
IGRP: Islet-specific glucose-6-phosphatase catalytic subunit-related protein
IBS: Irritable bowel syndrome
IBD: Inflammatory bowel disease
I*κ*B: Inhibitor of nuclear factor *κ*B (NF-*κ*B)
IL: Interleukin
IL-1R: Interleukin-1 receptor
IRF: Interferon regulatory factor
IRS-1: Insulin receptor substrate-1
JNK: c-Jun N-terminal kinase
KO: Knockout
KRas: Kirsten rat sarcoma viral oncogene homolog
LPS: Lipopolysaccharides
MafA: v-maf avian musculoaponeurotic fibrosarcoma oncogene homolog A
MAMP: Microbe-associated molecular pattern
MAPK: Mitogen-activated protein kinase
MyD88: Myeloid differentiation primary response gene 88
NET: Neuroendocrine tumor
NF-*κ*B: Nuclear factor *κ*B
NLR: Nucleotide-binding oligomerization domain-leucine-rich repeat; NOD-like receptor
NLRC: Nucleotide-binding oligomerization domain- (NOD-) like receptor with a CARD (caspase recruitment domain); NOD-leucine-rich repeat family with a CARD domain
NLRP: Nucleotide-binding domain, leucine-rich repeat-containing family, pyrin domain-containing protein
NOD: Nonobese diabetic
NOD1: Nucleotide oligomerization domain 1
NOD2: Nucleotide oligomerization domain 2
PAC: Pancreatic adenocarcinoma
PDAC: Pancreatic ductal adenocarcinoma
PRR: Pattern recognition receptor
PDX-1: Pancreatic and duodenal homeobox 1
PPAR-*α*: Peroxisome proliferator-activated receptor-*α*
ssRNA: Single-stranded RNA
T1D: Type 1 diabetes mellitus
T2D: Type 2 diabetes mellitus
TGF-*β*: Transforming growth factor beta
Th2: T helper type 2
TLR: Toll-like receptor
TNF-*α*: Tumor necrosis factor alpha
T_REG_: Regulatory T cells
TRIF: Toll/IL-1 receptor- (TIR-) domain-containing adapter-inducing interferon-*β*
WT: Wild-type
ZnT8: Zinc transporter 8.
